# COVID-19 vaccination effectiveness in the population of Friuli Venezia Giulia, North-East Italy. Control of bias associated with divergent compliance to policies in a test-negative case-control study

**DOI:** 10.1186/s12889-023-17244-9

**Published:** 2023-12-11

**Authors:** Valentina Rosolen, Federico Turoldo, Gianna Zamaro, Flavio Del Bianco, Patrizio Pezzotti, Luigi Castriotta, Fabio Barbone

**Affiliations:** 1 Central Directorate for Health, Social Policies and Disability, Friuli Venezia Giulia Region, Via Cassa Di Risparmio 10, Trieste, 34121 Italy; 2 Department of Medicine, Surgery and Health Sciences, University of Trieste, Strada di Fiume 447, Trieste, 34149 Italy; 3 Prevention Technical Platform, “AS FO” Western Friuli Health Authority, Via della Vecchia Ceramica 1, Pordenone, 33170 Italy; 4 Department of Infectious Diseases, National Institute of Health (ISS), Viale Regina Elena 299, Rome, 00161 Italy; 5 Institute of Hygiene and Evaluative Epidemiology, Friuli Centrale University Health Authority, Via Colugna 50, Udine, 33100 Italy

**Keywords:** COVID-19, Vaccine effectiveness, Mediation analysis, Alfa variant, Delta variant, Omicron variant, Selection Bias

## Abstract

**Background:**

Vaccine effectiveness (VE) studies consolidate knowledge of real-world effectiveness in different contexts. However, methodological issues may undermine their conclusions: to assess the VE against COVID-19 within the Italian population, a specific threat to validity is related to the consequences of divergent compliance to the Green Pass policy.

**Methods:**

To address this challenge we conducted a test negative case-control (TNCC) study and multiple sensitivity analysis among residents aged ≥ 12 in Friuli Venezia Giulia Region (FVG), North-east Italy, from February 1, 2021 to March 31, 2022. Information regarding 211,437 cases of COVID-19 infection and 845,748 matched controls was obtained from the regional computerized health database. The investigation considered: COVID-19 infection, hospitalization, and death. Multiple conditional logistic regressions adjusted for covariates were performed and VE was estimated as (1-OR COVID-19_vaccinated vs. unvaccinated_)x100. Mediation analyses were carried out to offset potential collider variables, particularly, the number of swabs performed after the introduction of pandemic restrictions.

**Results:**

Full-cycle VE against infection decreased from 96% (95% CI: 96, 97) in the Alpha period to 43% (95% CI: 42, 45) in the Omicron period. Booster dose raised the protection in Omicron period to 67% (95% CI: 66, 67). Against the evasive Omicron variant, the protection of the booster dose was 87% (95% CI: 83, 90) for hospitalization and 90% (95% CI: 82, 95) for death. The number of swabs performed was included as a covariate in the adjustments, and the mediation analysis confirmed that it was a strong mediator between vaccination and COVID-19-related outcomes.

**Conclusions:**

The study suggests that, under similar TNCC settings, mediation analysis and adjustment for number of diagnostic tests should be included, as an effective approach to the challenge of differential testing behavior that may determine substantial selection bias. This correction allowed us to align with results from other studies that show how full-cycle VE against infection was initially high but decreased over time by variant circulation, counterbalanced by booster dose that raised protection across variants and outcome severity.

**Supplementary Information:**

The online version contains supplementary material available at 10.1186/s12889-023-17244-9.

## Background

After randomized clinical trials (RCT) had demonstrated a high level of efficacy of COVID-19 vaccines in protecting against symptomatic infection, the European Medicines Agency (EMA) and the Italian national authority authorized their use and vaccines became available in Italy for priority groups on December 27, 2020 [1].

Since then, the question of vaccine effectiveness (VE) in real-world settings has been widely addressed [2]. Relevant VE topics include how to monitor the VE in the context of clinical factors not completely assessed by clinical trials, to investigate different outcomes, to determine the duration of the protection, and the VE against new Variants Of Concern (VOCs). The constant assessment of the real-world performance of these vaccines is required to justify health policies that must be adopted in different settings. However, observational VE studies, unlike RCT, have to overcome major methodological challenges, particularly in avoiding confounding and selection bias [3]. Therefore, several strategies of bias minimization have to be taken into consideration, as summarized in World Health Organization (WHO) guidance publications [4, 5].

In the pursuit of assessing COVID-19 VE in Italy, a specific threat to validity is related to the consequences of divergent population compliance to policies that have been adopted over the pandemic. In particular, the EU Regulation 2021/953 [6] granted the “Green-Pass” digital certificate to COVID-19 vaccinated, recovered or recently tested negative subjects (within 48 h), thus influencing citizens’ choices on numerous activities. Since the implementation of this European Regulation allowed each member state to introduce its own rules, its consequences varied by country [7]. In Italy, between October 15, 2021 and March 31, 2022 access to public spaces, means of transportation and workplaces [8–10] were subject to Green-Pass possession. Therefore, in that period, people’s daily life was regulated by clinical status, COVID-19 infection, contact with infected subjects, vaccination and proof of COVID-19 negative status.

To estimate VE against COVID-19 infection, hospitalization and death, we conducted a case-control study in the population > 12 years old of Region Friuli Venezia Giulia (FVG), North-east Italy. To mitigate potential confounding and biases, especially health seeking behavior bias and collider bias [11] that might have been originated by an heterogeneous adherence to Green Pass regulation, we chose a test-negative, case-control (TNCC) study [4, 12] reinforced by a series of sensitivity analyses.

## Methods

### Study population

As of January 1, 2022, the population of FVG Region was 1 194 647, accounting for 2.0% of the Italian population [13]. Regions in Italy are administrative and political entities that have authority on public health: during the pandemic they were in charge of organizing and implementing vaccination and testing strategies at the local level, and also of collecting and reporting health surveillance data [14]. The study population included subjects: (a) who had received at least one valid result for an antigenic or molecular SARS-CoV-2 virus swab at a public or private facility authorized by the Central Directorate of Health, Social Policies and Disability of FVG between February 1, 2021 and March 31, 2022, (b) who were residents in FVG at the date of swab collection and (c) who had been residents in FVG also from January 1, 2019 to February 1, 2021. The latter criterion was added to assess subjects’ comorbidities in the 2 years preceding the beginning of the study. Subjects with previous COVID-19 positivity were excluded.

### Source of COVID-19 data

The anonymized regional health database allows individual computerized linkage of comorbidity, diagnostic, treatment and outcomes information using a unique key. Specifically, we linked demographic data with swabs collected and COVID-19 vaccine doses, COVID-19-related hospitalizations and deaths, pre-COVID medications and hospital admissions. Certain swabs were excluded from the analysis: i.e., (i) swabs of children under age 12 years; (ii) negative swabs of subjects who turned out to be positive in the following 15 days; (iii) swabs of subjects whose vaccination status against COVID-19 was undefined; (iv) salivary molecular swabs collected before May 14, 2021 and antigenic swabs with positive results carried out in pharmacies before December 31, 2021, in accordance with the surveillance policy of regional and national authorities [15, 16]; (v) all the swabs following the first positive one.

### Study design

In this study population, we conducted a TNCC to estimate the COVID-19 VE against COVID-19 infection, hospitalization and death. For each case, four controls were randomly selected without replacement. Matching was based only on the date of swab collection (index date) while age, sex, province of residence and comorbidities score were included in models as covariates to avoid overmatching and control for confounding [17–19].

To estimate COVID-19 VE against infection (TNCC-INF), the cases were subjects with a first positive swab. The controls were randomly sampled among negative subjects matched by date of swab collection of the case.

Concerning COVID-19-related hospitalizations (TNCC-HOSP), the cases were swab positive subjects who were hospitalized in a COVID-19 ward, with a date of hospital admission within 30 days preceding the swab or 90 days following the swab date and the controls were sampled among swab negative, not hospitalized subjects.

The rationale behind the choice of these time cut-offs is explained as follow: according to the Italian Ministry of Health, any positive swab taken more than 90 days after the previous one defines a case of “re-infection”, and is considered as a different infectious event [20]. Furthermore, in order to include all hospitalizations of symptomatic subjects to COVID-19 wards with an admission date prior to that of the positive swab, we considered a period of 30 days as adequate.

To evaluate VE against COVID-19-related death (TNCC-DEATH), the cases were defined as subjects who died within 45 days following testing positive, while the controls were sampled among swab negative subjects at the date of death of the matched case.

Data from the Italian National Institute of Statistics show that 89% of COVID-19 related death occur within 30 days from the diagnosis, so the 45-days cut-off was chosen as adequate to include the vast majority of COVID-19 related deaths [21].

Our study covers periods characterized by different prevalent SARS-CoV-2 variants. They included: Alpha (Pango lineage B.1.1.7), Delta (B.1.617.2) and Omicron (B.1.1.529). VOC’s prevalence was estimated by the Italian National Institute of Health (ISS) which ran periodic regional surveys randomly sequencing a sample of daily swabs [22].

As mentioned above, the Italian application of the Green Pass legislation also determined a breaking time in the Italian COVID-19 epidemics, introducing the Green-Pass requirement for work and other daily-life activities [8–10]. In particular, the introduction of restrictions in the workplace forced many unvaccinated healthy workers to undergo several swabs every week in order to work, thus affecting the selection in our study. For these reasons our study hypothesis was tested separately in the following four sub-periods: (i) from February 1, 2021 to May 17, 2021 with prevalence of variant Alpha (PERIOD 1); (ii) from May 18, 2021 to October 14, 2021 with the transition and prevalence of variant Delta and before the introduction of Green-Pass requirement in workplaces (PERIOD 2); (iii) from October 15, 2021 to December 19, 2021 with prevalence of variant Delta and after the introduction of Green-Pass requirement in workplaces (PERIOD 3); (iv) from December 20, 2021 to March 31, 2022 with the transition and prevalence of variant Omicron (PERIOD 4).

### Intervention

The national vaccination campaign began on December 27, 2020; by February 1, 2021 approximately 1,5% of the regional population > 12 years old had received its second jab [23] (Figure S1). The following COVID-19 vaccines were used in FVG, during the study period, by date: Comirnaty (BNT162b2), from February 1, 2021 to March 31, 2022, full cycle and booster dose; Vaxzevria (ChAdOx1-S), from February 17, 2021 to October 21, 2021, full cycle; Janssen (Ad26.COV2.S), from April 29, 2021 to March 8, 2022, full cycle; Spikevax (mRNA-1273), from February 13, 2021 to March 31, 2022, full cycle and booster dose; Nuvaxovid (NVX-CoV2373), from March 3, 2022 to March 31, 2022, full cycle. Subjects’ vaccination status against COVID-19, assessed on the date of swab’s collection, was defined as follows: (i) None: a person who had not taken any dose or who had taken the first dose and less than 15 days had passed since the first dose; (ii) Partly vaccinated: a person who had received the first dose and at least 15 days had passed since the first dose or a person who had received the second dose but less than 14 days had passed since the second dose; (iii) Full cycle: a person who received the first dose of Janssen or the second dose of another vaccine and at least 15 days had passed since the first Janssen dose or the second dose of another vaccine; (iv) Booster dose: a person who had received the booster dose and at least 15 days had passed since the booster dose.

### Covariates

Gender, age, province of residence in FVG at swab’s collection, comorbidities score of each participant and number of swabs performed in the index date were considered as covariates.

Comorbidity was assessed by the Multisource Comorbidity Score (MCS), combining data from administrative health sources of FVG. The MCS is a risk adjustment tool based on hospital discharge diagnoses and drug prescriptions that measures the one-year risk of death. The higher the score, the higher the one-year risk of death [24].

The number of swabs performed in the month of the index date was also considered as a covariate in PERIOD 3 and in PERIOD 4 because testing might have become a confounder or a mediator of the relationship between vaccination status and the study outcomes after the aforementioned legislation was introduced on October 15, 2021.

### Statistical analysis

We calculated frequency and percentage distribution of the main characteristics of the residents in FVG who were tested at least once in the study period and of cases and controls stratified by sub-period.

To estimate the association between COVID-19 vaccination and infection-related outcomes, simple and multiple conditional logistic regressions adjusted for covariates were performed. Odds Ratios (OR) and 95% confidence interval (95% CI) were estimated. COVID-19 VE was estimated as (1-OR_in COVID−19 vaccinated vs. unvaccinated_)x100. Since our choice privileged a design that estimated real-world VE in the actual mixed and waned population, we did not consider nor adjusted for the waning of vaccine protection.

In PERIOD 3 and PERIOD 4, we conducted stratified analysis to assess whether the estimated OR was modified by the number of swabs performed in the month of the index date. Furthermore, to better understand the mediation role of the latter variable (dichotomized in “2 swabs or more” versus “1 swab”), we conducted a mediation analysis between vaccination status (dichotomized in “At least 1 dose” versus “None”) and infection, hospitalization and death [25, 26]. In the TNCC-DEATH for both sub-period, 1000 bootstrap samples were used to estimate bias-corrected bootstrap confidence interval. Finally, in the mediation analysis model in the PERIOD 4 the variable age was considered continuous to achieve model convergence.

All the statistical analyses were performed using SAS (version 9.4 SAS Institute Inc., Cary, NC, USA).

## Results

### Population base

**Fig. 1:**
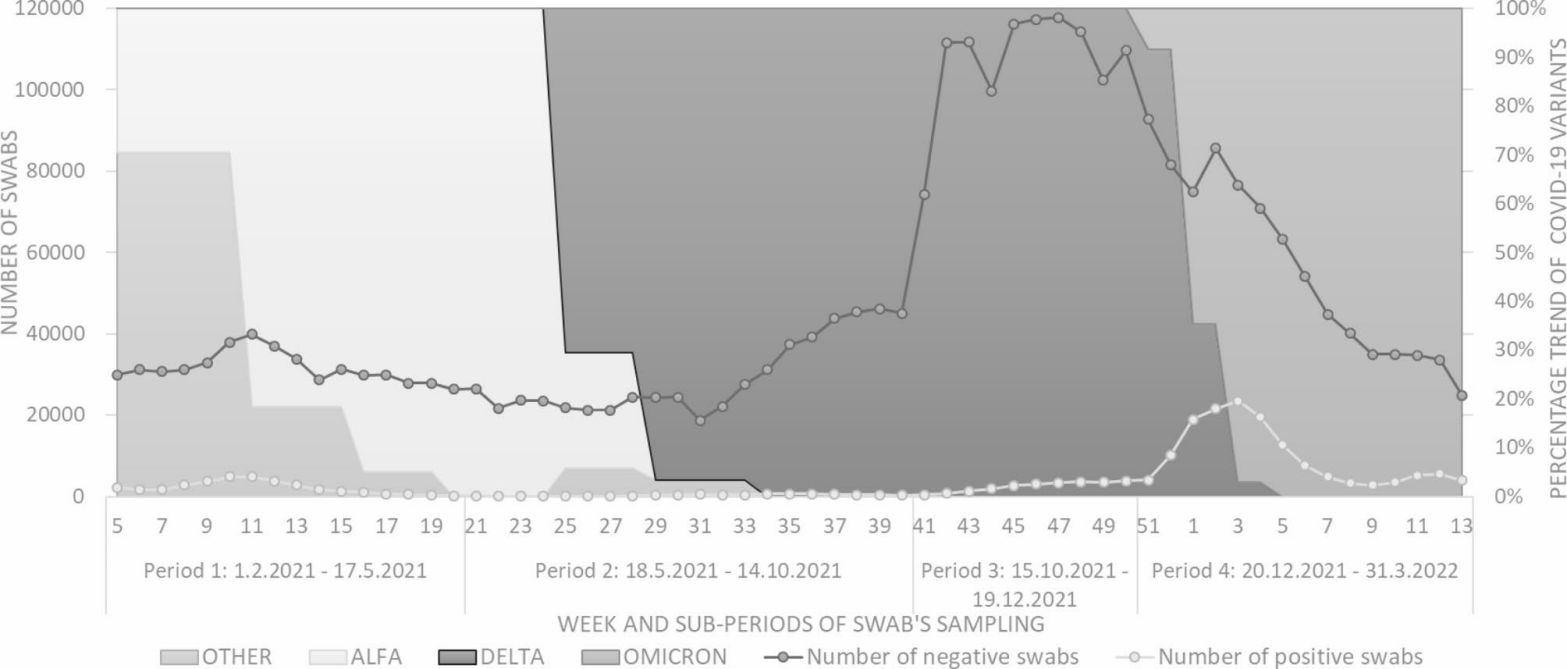
Frequency distribution of COVID-19 swabs tested in FVG in the study period by swab’s result The VOC’s prevalence in the region FVG was estimated by the National Institute of Health (ISS) during periodic surveys [22]

The main characteristics of the 627 982 subjects, aged 12 and over and residing in FVG with at least one swab in the whole study period are described in Table 1. Swabs sampled in this population base show an abrupt increase in the number of negative tests starting October 15, 2021, as a consequence of the introduction of Green Pass-related restrictions in workplaces (Fig. 1). A concurrent increase in the percentage of swabs taken by unvaccinated people is displayed in Figure S2.

**Table 1:** Characteristics of residents in FVG with at least one COVID-19 swab in the study period^a^

Characteristics	n	%
**Gender:**		
Female	323,730	51.6
Male	304,252	48.5
**Age groups (years)**:		
12–19	69,074	11.0
20–29	68,376	10.9
30–39	78,177	12.5
40–49	107,090	17.1
50–59	116,027	18.5
60–69	77,437	12.3
70–79	63,072	10.0
80–89	38,444	6.1
≥ 90	10,285	1.6
**Province of residence**:		
Gorizia	72,419	11.5
Pordenone	146,107	23.3
Trieste	129,781	20.7
Udine	279,675	44.5
**MCS** ^b^		
0	422,454	67.3
1–4	144,822	23.1
5–9	35,206	5.6
10–14	14,748	2.4
15–19	5689	0.9
≥ 20	5063	0.8

^a^Characteristics of residents in FVG, aged 12 or older, at the time of their first COVID-19 swab in the study period (1/2/2021-31/3/2022) is presented

^b^Multisource Comorbidity Score [24]

For each COVID-19-related outcome (infection, hospitalization and death), results are reported as follows:

(i) a table with the results of the multiple conditional logistic regression analysis for each of the four sub-periods considered in the study (for PERIOD 3 and 4 the multiple conditional logistic regressions were also adjusted for the number of swabs performed);
(ii) a figure with the results of the mediation analysis performed in the PERIOD 3 and 4;
(iii) the frequency’s and percentage distributions of population’s characteristics and further sensitive analysis included in the supplementary materials.

### Infection

**Fig. 2:**
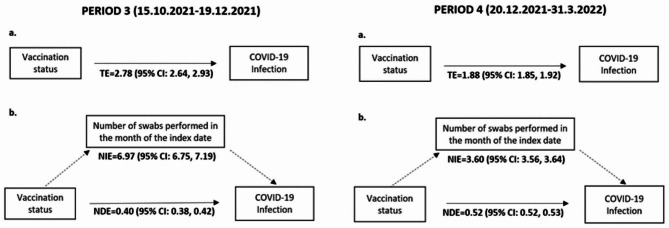
Mediation analysis of the “number of swabs performed” on the association between COVID-19 infection and vaccination status Path Diagram: **a**. Effect of vaccination status on the COVID-19 infection; **b**. Effect of vaccination status on the COVID-19 infection mediated by the number of swabs performed in the month of the index date The numbers represent Odds Ratios and their 95% confidence interval (95% CI) in the total, indirect or direct effect estimates in the PERIOD 3 and 4. TE = Total Effect; NIE = Natural Indirect Effect; NDE = Natural Direct Effect

In TNCC-INF, we identified 211 437 cases and 845 748 controls. The frequency and percentage distributions of the main characteristics of cases and controls are reported in Table S1. The corresponding results of multiple conditional logistic regression are displayed in Table 2: the full cycle VE against infection decreased from 96% (95% CI: 96, 97) in PERIOD 1 to 43% (95% CI: 42, 45) in PERIOD 4. Booster dose was able to rise the protection considerably. The results of mediation analysis (Fig. 2) in PERIOD 3, show that the total effect (TE), estimated by the OR, of vaccination status on COVID-19 infection was 2.78 (95% CI: 2.64, 2.93), but when the TE was decomposed into natural indirect effect (NIE) (OR: 6.97; 95% CI: 6.75, 7.19), and natural direct effect (NDE) (OR: 0.40; 95% CI: 0.38, 0.42), the effect was mainly mediated by the number of swabs performed in the month of the index date. In PERIOD 4 similar results were obtained: TE = 1.88 (95% CI: 1.85, 1.92), NIE = 3.60 (95% CI: 3.56, 3.64), NDE = 0.52 (95% CI: 0.52, 0.53). These results are consistent with those of the multiple conditional logistic regression stratified by the number of swabs performed in the month of the index date, shown in Table S2 and Table S3: the association described as the ORs between COVID-19 infection and vaccination status is confirmed in the different strata in both sub-periods.

**Table 2:** Multiple conditional logistic regression results by sub-periods. Outcome: COVID-19 infection

Vaccination status:	Cases (%)	Controls (%)	OR (95% CI)^a,c^	VE % (95% CI)^b,d^
**PERIOD 1** **(01.2.2021–17.5.2021):**				
None	32,730 (96.6)	96,854 (71.5)	1	-
Partly vaccinated	800 (2.4)	10,241 (7.6)	0.23 (0.22, 0.25)	77 (75, 78)
Full cycle	341 (1.0)	28,389 (21.0)	0.04 (0.03, 0.04)	96 (96, 97)
Booster dose	0 (0)	0 (0)	-	-
**PERIOD 2** **(18.5.2021–14.10.2021)**:				
None	4326 (66.9)	12,106 (46.8)	1	-
Partly vaccinated	418 (6.5)	1557 (6.0)	0.74 (0.66, 0.83)	26 (17, 34)
Full cycle	1717 (26.6)	12,181 (47.1)	0.45 (0.42, 0.48)	55 (52, 58)
Booster dose	0 (0)	0 (0)	-	-
**PERIOD 3** **(15.10.2021–19.12.2021)**:				
None	11,553 (48.1)	69,041 (71.8)	1	-
Partly vaccinated	407 (1.7)	1202 (1.3)	0.42 (0.35, 0.50)	58 (50, 65)
Full cycle	11,834 (49.2)	22,273 (23.2)	0.35 (0.33, 0.37)	65 (63, 67)
Booster dose	237 (1.0)	3608 (3.8)	0.06 (0.05, 0.07)	94 (93, 95)
**PERIOD 4** **(20.12.2021–31.3.2022)**:				
None	44,605 (30.3)	254,907 (43.3)	1	-
Partly vaccinated	3653 (2.5)	13,005 (2.2)	0.48 (0.46, 0.50)	52 (50, 54)
Full cycle	64,760 (44.0)	132,414 (22.5)	0.57 (0.55, 0.58)	43 (42, 45)
Booster dose	34,056 (23.2)	187,970 (32.0)	0.33 (0.33, 0.34)	67 (66, 67)

^a^ Odds Ratio (OR) and 95% confidence intervals (95% CI) adjusted for gender, classes of age, province of residence and Multisource Comorbidity Score in PERIOD1 and PERIOD2

^b^Percentage of Vaccination effectiveness and 95% confidence intervals (95% CI) adjusted for gender, classes of age, province of residence and Multisource Comorbidity Score Score in PERIOD1 and PERIOD2

^c^Odds Ratio (OR) and 95% confidence intervals (95% CI) adjusted for gender, classes of age, province of residence, Multisource Comorbidity Score and number of swabs performed in the month of the index date Score in PERIOD3 and PERIOD4

^d^Percentage of Vaccination Effectiveness and 95% Confidence Intervals (95% CI) adjusted for gender, classes of age, province of residence, Multisource Comorbidity Score and number of swabs performed in the month of the index date Score in PERIOD3 and PERIOD4

### Hospitalization

In the TNCC-HOSP, we identified 7867 cases and 31 468 controls. Frequency and percentage distributions of the main characteristics of cases and controls are presented in Table S4. The results of multiple conditional logistic regression, stratified by sub-periods, are presented in Table 3: compared to unvaccinated subjects, full cycle VE against COVID-19-related hospitalization was 97% (95% CI: 95, 98) and 84% (95% CI: 77, 89) in PERIOD 1 and 2, respectively. During PERIOD 3 and 4, the VE against hospitalization increased with increasing doses of vaccine. Particularly, VE of booster dose was 98% (95% CI: 95, 99) in PERIOD 3 and 87% (95% CI: 83, 90) in PERIOD 4.

**Fig. 3:**
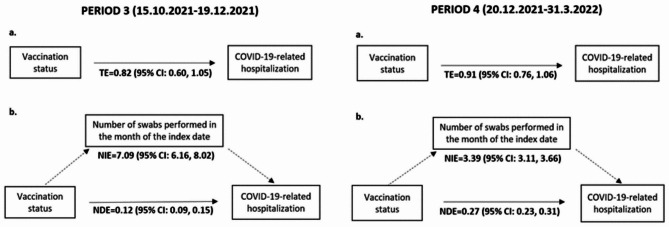
Mediation analysis of the “number of swabs performed” on the association between COVID-19-related hospitalization and vaccination status Path Diagram: **a**. Effect of vaccination status on the COVID-19-related hospitalization; **b**. Effect of vaccination status on the COVID-19-related hospitalization mediated by the number of swabs performed in the month of the index date The numbers represent Odds Ratios and their 95% confidence interval (95% CI) in the total, indirect or direct effect estimates in the PERIOD 3 and 4. TE = Total Effect; NIE = Natural Indirect Effect; NDE = Natural Direct Effect

The results of mediation analysis (Fig. 3) show that in PERIOD 3 the TE of vaccination status on hospitalization was 0.82 (95% CI: 0.60, 1.05), but when the TE was decomposed into NIE (OR: 7.09; 95% CI: 6.16, 8.02) and NDE (OR: 0.12; 95% CI: 0.09, 0.15), the effect was mainly mediated by the number of swabs performed in the month of the index date. In PERIOD 4 similar results were obtained: TE = 0.91 (95% CI: 0.76, 1.06), NIE = 3.39 (95% CI: 3.11, 3.66), NDE = 0.27 (95% CI: 0.23, 0.31). Consistently, multiple conditional logistic regression stratified by the number of swabs performed in the month of the index date (Table S5 and Table S6), suggests a strong association between COVID-19 hospitalization and vaccination for any dose of vaccine and the number of swabs performed. However, in some cases, the ORs were imprecise or could not be estimated due to a small number of observations in the different strata.

**Table 3:** Multiple conditional logistic regression results by sub-periods. Outcome: COVID-19-related hospitalization

Vaccination status	Cases (%)	Controls (%)	OR (95% CI)^a,c^	VE % (95% CI)^b,d^
**PERIOD 1** **(01.02.2021–17.05.2021):**				
None	3269 (96.2)	9707 (71.4)	1	-
Partly vaccinated	105 (3.1)	966 (7.1)	0.23 (0.18, 0.30)	76 (70, 82)
Full cycle	26 (0.8)	2927 (21.5)	0.03 (0.02, 0.05)	97 (95, 98)
Booster dose	0 (0.0)	0 (0.0)	-	-
**PERIOD 2** **(18.5.2021–14.10.2021)**:				
None	292 (68.1)	827 (48.2)	1	-
Partly vaccinated	15 (3.5)	102 (5.9)	0.24 (0.12, 0.47)	76 (53, 88)
Full cycle	122 (28.4)	787 (45.8)	0.16 (0.11, 0.23)	84 (77, 89)
Booster dose	0 (0.0)	0 (0.0)	-	-
**PERIOD 3** **(15.10.2021–19.12.2021)**:				
None	803 (54.0)	4364 (73.3)	1	-
Partly vaccinated	24 (1.6)	60 (1.0)	0.87 (0.28, 2.72)	13 (< 0, 72)
Full cycle	635 (42.7)	1333 (22.4	0.13 (0.09, 0.20)	87 (80, 91)
Booster dose	26 (1.8)	195 (3.3)	0.02 (0.01, 0.05)	98 (95, 99)
**PERIOD 4** **(20.12.2021–31.3.2022)**:				
None	931 (36.5)	4629 (45.4)	1	-
Partly vaccinated	79 (3.1)	224 (2.2)	0.80 (0.46, 1.37)	20 (< 0, 54)
Full cycle	810 (31.8)	2182 (21.4)	0.41 (0.33, 0.51)	59 (49, 66)
Booster dose	730 (28.6)	3165 (31.0)	0.13 (0.10, 0.17)	87 (83, 90)

^a^ Odds Ratio (OR) and 95% confidence intervals (95% CI) adjusted for gender, classes of age, province of residence and Multisource Comorbidity Score in PERIOD1 and PERIOD2

^b^Percentage of Vaccination effectiveness and 95% confidence intervals (95% CI) adjusted for gender, classes of age, province of residence and Multisource Comorbidity Score Score in PERIOD1 and PERIOD2

^c^Odds Ratio (OR) and 95% confidence intervals (95% CI) adjusted for gender, classes of age, province of residence, Multisource Comorbidity Score and number of swabs performed in the month of the index date Score in PERIOD3 and PERIOD4

^d^Percentage of Vaccination Effectiveness and 95% Confidence Intervals (95% CI) adjusted for gender, classes of age, province of residence, Multisource Comorbidity Score and number of swabs performed in the month of the index date Score in PERIOD3 and PERIOD4

### Death

**Fig. 4:**
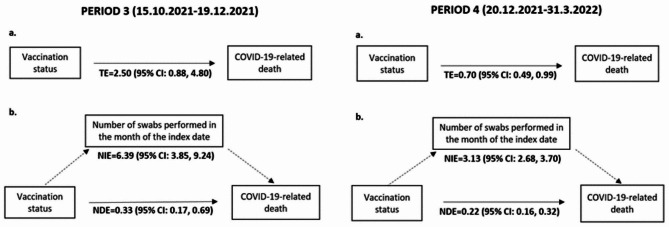
Mediation analysis of the “number of swabs performed” on the association between COVID-19-related death and vaccination status Path Diagram: **a**. Effect of vaccination status on the COVID-19-related death; **b**. Effect of vaccination status on the COVID-19-related death mediated by the number of swabs performed in the month of the index date The numbers represent Odds Ratios and their 95% confidence interval (95% CI) in the total, indirect or direct effect estimates in the PERIOD 3 and 4. TE = Total Effect; NIE = Natural Indirect Effect; NDE = Natural Direct Effect. One thousand bootstrap samples were used to estimate bias-corrected bootstrap confidence interval. In the model of PERIOD 4 the variable age was considered continuous to achieve model convergence

Analysis of the TNCC-DEATH study included 2040 cases and 8160 controls. The frequency and percentage distributions of the main characteristics of cases and controls are reported in Table S7. The corresponding results of multiple conditional logistic regression are presented in Table 4. During the four sub-periods, VE against death increased with increasing vaccine doses performed, although estimates, in some cases, were imprecise due to small number of observations in some levels of vaccination status. Anyway, the VE against death was steadily high, full cycle VE varying from 98% (95% CI: 94; 99) in PERIOD 1 to 63% (95% CI: 31, 80) in PERIOD 4. Booster dose in PERIOD 4 was able to rise the protection to 90% (95% CI: 82, 95). Figure 4 shows the results of mediation analysis conducted in PERIOD 3 and 4. In PERIOD 3, the TE of vaccination status on COVID-19-related death was 2.50 (95% CI: 0.88, 4.80), but when the TE was decomposed into NIE (OR: 6.39; 95% CI: 3.85, 9.24) and NDE (OR: 0.33; 95% CI: 0.17, 0.69), the effect was mainly mediated by the number of swabs performed in the month of the index date. Also during PERIOD 4, the effect of vaccination status on the COVID-19-related death is mediated by of number of swabs performed in the month of the index date: TE = 0.70 (95% CI: 0.49, 0.99), NIE = 3.13 (95% CI: 2.68, 3.70), NDE = 0.22 (95% CI: 0.16, 0.32). The multiple conditional logistic regression stratified by the number of swabs gives consistent results in the strata with 1 swab for both sub-period (Table S8 and Table S9). However, for the remaining strata the ORs are not presented due to the low number of observations.

**Table 4:** Multiple conditional logistic regression results by sub-periods. Outcome: COVID-19-related death

Vaccination status	Cases (%)	Controls (%)	OR (95% CI)^a,c^	VE % (95% CI)^b,d^
**PERIOD 1** **(01.02.2021–17.05.2021):**				
None	836 (91.4)	2518 (68.8)	1	-
Partly vaccinated	71 (7.8)	232 (6.3)	0.38 (0.24, 0.61)	62 (39, 76)
Full cycle	8 (0.9)	910 (24.9)	0.02 (0.01, 0.06)	98 (94; 99)
Booster dose	0 (0.0)	0 (0.0)	-	-
**PERIOD 2** **(18.5.2021–14.10.2021)**:				
None	28 (48.3)	98 (42.2)	1	-
Partly vaccinated	5 (8.6)	18 (7.8)	0.05 (< 0.001, 5.28)	95 (< 0, 99.9)
Full cycle	25 (43.1)	116 (50.0)	0.02 (0.001, 0.28)	98 (72, 99.99)
Booster dose	0 (0.0)	0 (0.0)	-	-
**PERIOD 3** **(15.10.2021–19.12.2021)**:				
None	131 (40.7)	942 (73.1)	1	-
Partly vaccinated	9 (2.8)	15 (1.2)	10.33 (< 0.001, > 999)	< 0 (< 0, > 99)
Full cycle	164 (50.9)	284 (22.1)	0.06 (0.004, 0.96)	94 (4, 99)
Booster dose	18 (5.6)	47 (3.6)	0.001 (< 0.001, 0.71)	99.9 (29, > 99)
**PERIOD 4** **(20.12.2021–31.3.2022)**:				
None	275 (36.9)	1340 (45.0)	1	-
Partly vaccinated	24 (3.2)	75 (2.5)	0.69 (0.17, 2.83)	31 (< 0, 83)
Full cycle	219 (29.4)	614 (20.6)	0.37 (0.20, 0.69)	63 (31, 80)
Booster dose	227 (30.5)	951 (31.9)	0.10 (0.05, 0.18)	90 (82, 95)

^a^ Odds Ratio (OR) and 95% confidence intervals (95% CI) adjusted for gender, classes of age, province of residence and Multisource Comorbidity Score in PERIOD1 and PERIOD2

^b^Percentage of Vaccination effectiveness and 95% confidence intervals (95% CI) adjusted for gender, classes of age, province of residence and Multisource Comorbidity Score Score in PERIOD1 and PERIOD2

^c^Odds Ratio (OR) and 95% confidence intervals (95% CI) adjusted for gender, classes of age, province of residence, Multisource Comorbidity Score and number of swabs performed in the month of the index date Score in PERIOD3 and PERIOD4

^d^Percentage of Vaccination Effectiveness and 95% Confidence Intervals (95% CI) adjusted for gender, classes of age, province of residence, Multisource Comorbidity Score and number of swabs performed in the month of the index date Score in PERIOD3 and PERIOD4

## Discussion

At the design stage of our study, we were concerned about the potential presence of selection bias as this is a major issue in all observational studies conducted in the field of VE. To mitigate this issue, we chose a Test-Negative design that confirmed, via testing, that controls were not infected at the time of case diagnosis. In addition, the TNCC approach controls for some health seeking behavior [4]. However, it has been demonstrated that in the Test-Negative design a source of selection bias could be represented by a collider variable related to the propensity to be included in the sample [11].

This was the situation in Italy between October 15, 2021 and March 31, 2022. During the period of the Green-pass requirement for workers, we observed that unvaccinated people had to undergo several tests every week in order to work. This was unrelated to other indications and to the health seeking behavior, and thus introduced a substantial difference in the testing process between vaccinated and unvaccinated, consequently affecting inclusion in the study. In fact, to access their workplace healthy unvaccinated subjects conducted many tests that resulted as negative, and therefore could be selected as controls.

Since such a bias was not intrinsically controlled by the study design, we adopted the abovementioned strategy to conduct for PERIOD 3 and PERIOD 4 adjustment and stratified analysis by the number of swabs performed in the month of the index date: the resulting estimates of VE are in accordance with most available literature on the topic. Also, the mediation analysis that was carried out confirmed the strong mediator role of the number of swabs performed in PERIOD 3 and 4 and the importance of such adjustments.

In the following subsections, separately for each outcome taken into consideration, we discuss the results obtained, provide a comparison of the estimated VE with available evidence, and argue the limitations of the study.

### Infection

In the Alpha period, our estimates of protection against infection align well with another Italian study which gave similar results [27], and with other TNCC studies [28]. In particular, one meta-analysis of TNCC studies [29] gave very similar results for the Moderna and Pfizer vaccine, which cover 88% of vaccinations in the FVG region [23]. A comparison with another meta-analysis [30], is available in Table S10. During the Delta phase, our results of PERIOD 2 and PERIOD 3 are quite different. The available evidence seems to align better with PERIOD 3, after the restrictions for unvaccinated were introduced [29–32]. This also suggests that the adjustment for the number of tests undergone by participants reduced the bias in PERIOD 3 and gave result that are sufficiently externally valid. During PERIOD 4, our results are rather consistent with the evidence from the Italian National Institute of Health (ISS) [33] and other TNCC studies [34, 35].

Stratified results (Table S2 and Table S3) confirmed a strong protective effect of vaccination for any dose of vaccine and number of swabs.

The corresponding mediation analysis was able to demonstrate further a strong protective effect of vaccination with the concurrent indirect effect of testing. In fact, the variable related to the testing behavior is associated with higher odds of infection, as shown in the NIE (ORs: 6.97 and 3.60), and had not been taken into account, this effect would have biased considerably the analysis: therefore, this justifies the adjustment for the number of swabs performed in PERIOD 3 and PERIOD 4. When interpreting this result, we have to bear in mind that higher odds of infection associated with the number of tests performed is not only related to the selection bias, but might reflect also cumulative risk-taking behaviours, as people who choose not to get vaccinated (and will be likely be tested more often due to requirements in place) might also choose not to engage in non-pharmaceutical interventions (NPIs).

The protective effect of the vaccine is made visible in the NDE of the mediation analysis (ORs: 0.40 and 0.52), which represents the effect of the independent variable (dichotomized vaccination status) on the dependent variable (COVID-19 outcome) that is not explained by the mediator, or, in other words, if the mediator was not present.

Potential limitations of the results presented include the effect of previous infection not reported in clinical records: some subject might have developed an immunity which was not documented, and this is especially true in the earlier pandemic stages. If unvaccinated subjects were more prone to develop unrecorded infections, this could underestimate the VE. Differential exposure to risk could also result in spuriously altered VE estimates.

Furthermore, multiple sources of differential health seeking behavior could have been present, for example individuals with vaccination side effects might be more likely to seek medical care and testing, even if the design of the study attempted to tackle this aspect in an unbiased way.

### Hospitalization

The main methodological challenges and approach of the TNCC-INF also apply to severe outcomes. As shown in Table S10, regarding hospitalization, our results are consistent with the meta-analysis by Shao et al., since for all VOCs the 95% CIs largely overlap [30]; in PERIOD 1 our results align well also with local evidence [27]. In the two Delta periods, the difference between full cycle estimates is small and both are consistent with local evidence from Italy and with other European studies [31, 32, 36], and this is also for true for estimates in PERIOD 4 [37, 38].

Results for VE of partly vaccinated were limited by the small number of corresponding strata, as seen in Table S4. Also the stratified analysis available in Tables S5 and S6 are affected by the small numbers, and achieve statistical significance mainly in the strata relative to 1 swab performed in the month of the index date.

In the mediation analyses, both TE of PERIOD 3 and PERIOD 4 suggest an overall protective effect, as a combination of both the direct and indirect effects, even if they are not statistically significant. The protective effect is much more evident after the mediator effect is controlled for in the NDE (ORs: 0.12 and 0.27). This confirms that testing behavior plays a crucial role in the overall relationship between vaccination status and the outcome, and needs to be considered when assessing the protective effect of vaccination also regarding hospitalization.

Together with the low numbers of some strata, additional limitations are attributable to factors similar to those discussed in the previous paragraph regarding infections.

### Death

Overall, COVID-19 vaccines’ VE was high against death, although the interpretation of results should be cautious due to small numbers in some strata, especially for partial and booster dose VE. Also the multiple conditional logistic regression stratified by number of swabs was largely affected by sparse data.

Keeping this limitation in mind, 95%CI of our estimates largely overlap with the meta-analysis by Shao et al. [30] in every period taken into consideration (Table S10). The TNCC study by Castillo et al. also has similar results for Delta and Omicron [37].

In the mediation analysis, the TE in PERIOD 4 shows, overall, that vaccine status is associated with lower odds of death. The testing behaviour-related variable nonetheless acts as a strong mediator between vaccine status and death, and after it is controlled for, as shown in the NDE, a higher protective effect of vaccine status against death is revealed in both PERIOD 3 and PERIOD 4 (ORs: 0.33 and 0.22).

The definition of cases that we applied to COVID-19-related deaths employed a broad time-cut-off, including subjects who died within 45 days following testing positive. Therefore, it is possible that other clinical factors could have had time to develop and contribute to the patient’s death as well as COVID-19.

## Conclusions

In conclusion, the study suggests that due to the complexity of demonstrating VE in this real-world setting for the potential biases inherent in observational investigations, even under a TNCC design, when differential testing behavior is present determining substantial selection bias, mediation analysis and adjustment for number of diagnostic testing should be included. This correction allowed us to align with results from other studies that show how full-cycle VE against infection was initially high but decreased consistently over time by variant circulation, counterbalanced by booster dose which was able to increase protection in every period taken into consideration. A highly effective protection given by COVID-19 vaccines was also demonstrated against hospitalization and death.

### Electronic supplementary material

Below is the link to the electronic supplementary material.

**Supplementary Material 1: Figure S1**. Trend of vaccination coverage in the population of FVG. **Figure S2**. Percentage distribution of COVID-19 swabs taken in Friuli Venezia Giulia Region (1/2/2021-31/3/2022), by week, sub-period, and COVID-19 vaccination status. **Table S1**. Frequency and percentage distribution of cases and controls characteristics by sub-period. Outcome: COVID-19 Infection. **Table S2**. Frequency and percentage distribution of vaccination status in cases and controls, and multiple conditional logistic regression results stratified by number of swabs performed in the month of the index date. Outcome: COVID-19 Infection, PERIOD 3 (15/10/2021-19/12/2021). **Table S3**. Frequency and percentage distribution of vaccination status in cases and controls, and multiple conditional logistic regression results stratified by number of swabs performed in the month of the index date. Outcome: COVID-19 infection, PERIOD 4 (20/12/2021-31/3/2022). **Table S4**. Frequency and percentage distribution of cases and controls characteristics by sub-periods. Outcome: COVID-19-related hospitalization. **Table S5**. Frequency and percentage distribution of vaccination status in cases and controls, and multiple conditional logistic regression results stratified by number of swabs performed in the month of the index date. Outcome: COVID-19-related hospitalization, PERIOD 3 (15/10/2021-19/12/2021). **Table S6**. Frequency and percentage distribution of vaccination status in cases and controls, and multiple conditional logistic regression results stratified by number of swabs performed in the month of the index date. Outcome: COVID-19-related hospitalization, PERIOD 4 (20/12/2021-31/3/2022). **Table S7**. Frequency and percentage distribution of cases and controls characteristics by sub-periods. Outcome: COVID-19-related death. **Table S8**. Frequency and percentage distribution of vaccination status in cases and controls, and multiple conditional logistic regression results stratified by number of swabs performed in the month of the index date. Outcome: COVID-19-related death, PERIOD 3 (15/10/2021-19/12/2021). **Table S9**. Frequency and percentage distribution of vaccination status in cases and controls, and multiple conditional logistic regression results stratified by number of swabs performed in the month of the index date. Outcome: COVID-19-related death, PERIOD 4 (20/12/2021-31/3/2022). **Table S10**. Comparison of our results with the meta-analysis from Shao et al. [30].

## Data Availability

The data underlying this article will be shared on reasonable request to the corresponding author.

## List of abbreviations

CI: Confidence Interval;
COVID-19: Coronavirus disease 2019
FVG: Friuli Venezia-Giulia;
MCS: Multisource Comorbidity Score;
NDE: Natural Direct Effect;
NIE: Natural Indirect Effect;
NPIs: Non-Pharmaceutical Interventions;
OR: Odds Ratio;
RCT: Randomized Clinical Trial;
TE: Total Effect;
TNCC: Test-Negative Case Control;
VE: Vaccine effectiveness;
VOC: Variant Of Concern;
WHO: World Health Organization;
