# Supplementary material for: COVID-19 vaccination effectiveness in the population of Friuli Venezia Giulia, North-East Italy. Control of bias associated with divergent compliance to policies in a test-negative case-control study
Source: BMC Public Health. 2023 Dec 11;23:2476. doi: 10.1186/s12889-023-17244-9 (PMC10714502; doi:10.1186/s12889-023-17244-9)
Supplement: Supplementary file 1 — Supplementary Material 1: Figure S1. Trend of vaccination coverage in the population of FVG. Figure S2. Percentage distribution of COVID-19 swabs taken in Friuli Venezia Giulia Region (1/2/2021-31/3/2022), by week, sub-period, and COVID-19 vaccination status. Table S1. Frequency and percentage distribution of cases and controls characteristics by sub-period. Outcome: COVID-19 Infection. Table S2. Frequency and percentage distribution of vaccination status in cases and controls, and multiple conditional logistic regression results stratified by number of swabs performed in the month of the index date. Outcome: COVID-19 Infection, PERIOD 3 (15/10/2021-19/12/2021). Table S3. Frequency and percentage distribution of vaccination status in cases and controls, and multiple conditional logistic regression results stratified by number of swabs performed in the month of the index date. Outcome: COVID-19 infection, PERIOD 4 (20/12/2021-31/3/2022). Table S4. Frequency and percentage distribution of cases and controls characteristics by sub-periods. Outcome: COVID-19-related hospitalization. Table S5. Frequency and percentage distribution of vaccination status in cases and controls, and multiple conditional logistic regression results stratified by number of swabs performed in the month of the index date. Outcome: COVID-19-related hospitalization, PERIOD 3 (15/10/2021-19/12/2021). Table S6. Frequency and percentage distribution of vaccination status in cases and controls, and multiple conditional logistic regression results stratified by number of swabs performed in the month of the index date. Outcome: COVID-19-related hospitalization, PERIOD 4 (20/12/2021-31/3/2022). Table S7. Frequency and percentage distribution of cases and controls characteristics by sub-periods. Outcome: COVID-19-related death. Table S8. Frequency and percentage distribution of vaccination status in cases and controls, and multiple conditional logistic regression results stratified by number of swabs perf [file 12889_2023_17244_MOESM1_ESM.docx]

**Supplementary Materials**

**Figure S1. Trend of vaccination coverage in the population of FVG.**

1. At least one dose=first doses (including Janssen) + vaccination after previous infection.
2. Complete cycle=single-dose Janssen + second dose for two-dose regimen vaccines + vaccination after previous infection.

Data extracted 15/4/2022 [23]. The denominator of this graph is the population of FVG>12 as reported by the Italian Institute of Statistics (ISTAT) in 2021 [13]. The 14 days necessary for immunity development are not taken into consideration.

**Figure S2. Percentage distribution of COVID-19 swabs taken in Friuli Venezia Giulia Region (1/2/2021 – 31/3/2022), by week, sub-period, and COVID-19 vaccination status.**

**Table S1. Frequency and percentage distribution of cases and controls characteristics by sub-period. Outcome: COVID-19 Infection.**

|  | **PERIOD 1** | |  | **PERIOD 2** | |  | **PERIOD 3** | |  | **PERIOD 4** | |
| --- | --- | --- | --- | --- | --- | --- | --- | --- | --- | --- | --- |
|  | **01.02.2021 - 17.05.2021** | |  | **18.5.2021-14.10.2021** | |  | **15.10.2021-19.12.2021** | |  | **20.12.2021-31.3.2022** | |
|  | **Cases** | **Controls** |  | **Cases** | **Controls** |  | **Cases** | **Controls** |  | **Cases** | **Controls** |
|  | **(n=33871)** | **(n=135484)** |  | **(n=6461)** | **(n=25844)** |  | **(n=24031)** | **(n=96124)** |  | **(n=147074)** | **(n=588296)** |
| **Characteristics** | **n (%)** | **n (%)** |  | **n (%)** | **n (%)** |  | **n (%)** | **n (%)** |  | **n (%)** | **n (%)** |
| **Gender:** |  |  |  |  |  |  |  |  |  |  |  |
| Female | 16840 (49.7) | 79102 (58.4) |  | 3256 (50.4) | 15046 (58.2) |  | 12418 (51.7) | 49495 (51.5) |  | 77142 (52.5) | 320516 (54.5) |
| Male | 17031 (50.3) | 56382 (41.6) |  | 3205 (49.6) | 10798 (41.8) |  | 11613 (48.3) | 46629 (48.5) |  | 69932 (47.6) | 267780 (45.5) |
| **Age groups (years):** |  |  |  |  |  |  |  |  |  |  |  |
| 12-19 | 2802 (8.3) | 7517 (5.6) |  | 1057 (16.4) | 2633 (10.2) |  | 2630 (10.9) | 10092 (10.5) |  | 18958 (12.9) | 49168 (8.4) |
| 20-29 | 3359 (9.9) | 13918 (10.3) |  | 975 (15.1) | 2820 (10.9) |  | 2065 (8.6) | 10282 (10.7) |  | 18469 (12.6) | 60017 (10.2) |
| 30-39 | 3923 (11.6) | 19170 (14.2) |  | 905 (14.0) | 3527 (13.7) |  | 3024 (12.6) | 15261 (15.9) |  | 22279 (15.2) | 88562 (15.1) |
| 40-49 | 6075 (17.9) | 27828 (20.6) |  | 1119 (17.3) | 4716 (18.3) |  | 4966 (20.7) | 23075 (24.0) |  | 29785 (20.3) | 133497 (22.7) |
| 50-59 | 6876 (20.3) | 30694 (22.7) |  | 1091 (16.9) | 5437 (21.0) |  | 4711 (19.6) | 23423 (24.4) |  | 27922 (19.0) | 129116 (22.0) |
| 60-69 | 4444 (13.1) | 14518 (10.7) |  | 570 (8.8) | 2544 (9.8) |  | 2777 (11.6) | 7866 (8.2) |  | 14574 (9.9) | 59939 (10.2) |
| 70-79 | 3725 (11.0) | 10292 (7.6) |  | 378 (5.9) | 1814 (7.0) |  | 2175 (9.1) | 3193 (3.3) |  | 9231 (6.3) | 34419 (5.9) |
| 80-89 | 2040 (6.0) | 8424 (6.2) |  | 261 (4.0) | 1747 (6.8) |  | 1289 (5.4) | 2192 (2.3) |  | 4530 (3.1) | 25367 (4.3) |
| ≥90 | 627 (1.9) | 3123 (2.3) |  | 105 (1.6) | 606 (2.3) |  | 394 (1.6) | 740 (0.8) |  | 1326 (0.9) | 8211 (1.4) |
| **Province of residence:** |  |  |  |  |  |  |  |  |  |  |  |
| Gorizia | 4139 (12.2) | 18035 (13.3) |  | 583 (9.0) | 3131 (12.1) |  | 3058 (12.7) | 10835 (11.3) |  | 15116 (10.3) | 66214 (11.3) |
| Pordenone | 4640 (13.7) | 18191 (13.4) |  | 1706 (26.4) | 4212 (16.3) |  | 4848 (20.2) | 17016 (17.7) |  | 41382 (28.1) | 97344 (16.6) |
| Trieste | 6349 (18.7) | 32460 (24.0) |  | 2028 (31.4) | 6861 (26.6) |  | 9155 (38.1) | 21846 (22.7) |  | 28816 (19.6) | 126937 (21.6) |
| Udine | 18742 (55.3) | 66798 (49.3) |  | 2144 (33.2) | 11640 (45.0) |  | 6970 (29.0) | 46427 (48.3) |  | 61760 (42.0) | 297801 (50.6) |
| **MCS**^a^**:** |  |  |  |  |  |  |  |  |  |  |  |
| 0 | 22062 (65.1) | 85792 (63.3) |  | 4705 (72.8) | 16949 (65.6) |  | 16773 (69.8) | 72469 (75.4) |  | 107021 (72.8) | 413779 (70.3) |
| 1-4 | 8290 (24.5) | 30992 (22.9) |  | 1337 (20.7) | 5691 (22.0) |  | 5347 (22.3) | 18629 (19.4) |  | 31585 (21.5) | 126674 (21.5) |
| 5-9 | 2123 (6.3) | 8610 (6.4) |  | 264 (4.1) | 1663 (6.4) |  | 1177 (4.9) | 2761 (2.9) |  | 5179 (3.5) | 26486 (4.5) |
| 10-14 | 787 (2.3) | 4803 (3.6) |  | 97 (1.5) | 809 (3.1) |  | 452 (1.9) | 1268 (1.3) |  | 2003 (1.4) | 12215 (2.1) |
| 15-19 | 306 (0.9) | 2351 (1.7) |  | 28 (0.4) | 364 (1.4) |  | 168 (0.7) | 511 (0.5) |  | 728 (0.5) | 4620 (0.8) |
| ≥20 | 303 (0.9) | 2936 (2.2) |  | 30 (0.5) | 368 (1.4) |  | 114 (0.5) | 486 (0.5) |  | 558 (0.4) | 4522 (0.8) |
| **Vaccination status:** |  |  |  |  |  |  |  |  |  |  |  |
| None | 32730 (96.6) | 96854 (71.5) |  | 4326 (667.0) | 12106 (46.8) |  | 11553 (48.1) | 69041 (71.8) |  | 44605 (30.3) | 254907 (43.3) |
| Partly vaccinated | 800 (2.4) | 10241 (7.6) |  | 418 (6.5) | 1557 (6.0) |  | 407 (1.7) | 1202 (1.3) |  | 3653 (2.5) | 13005 (2.2) |
| Full cycle | 341 (1.0) | 28389 (21.0) |  | 1717 (26.6) | 12181 (47.1) |  | 11834 (49.2) | 22273 (23.2) |  | 64760 (44.0) | 132414 (22.5) |
| Booster dose | 0 (0) | 0 (0) |  | 0 (0) | 0 (0) |  | 237 (1.0) | 3608 (3.8) |  | 34056 (23.2) | 187970 (32.0) |
| **Swabs in index-date’s month^b,c^:** |  |  |  |  |  |  |  |  |  |  |  |
| 1 | 33229 (98.1) | 62095 (45.8) |  | 6284 (97.3) | 11730 (45.4) |  | 22192 (92.4) | 15443 (16.1) |  | 135524 (92.2) | 159616 (27.1) |
| 2 | 541 (1.6) | 33653 (24.8) |  | 160 (2.5) | 6007 (23.2) |  | 1037 (4.3) | 10664 (11.1) |  | 6531 (4.4) | 97474 (16.6) |
| ≥3 | 101 (0.3) | 39736 (29.3) |  | 17 (0.3) | 8107 (31.4) |  | 802 (3.3) | 70017 (72.8) |  | 5019 (3.4) | 331205 (56.3) |
| ^a^Multisource Comorbidity Score.  ^b^Number of swabs performed in the month of index date  ^c^Case’s date of sampling matched with control’s date of sampling. | | | | | | | | | | | |

**Table S2. Frequency and percentage distribution of vaccination status in cases and controls, and multiple conditional logistic regression results stratified by number of swabs performed in the month of the index date. Outcome: COVID-19 Infection, PERIOD 3 (15/10/2021-19/12/2021).**

|  | **Number of swabs performed in the month of index date:** | | | | | | | | |
| --- | --- | --- | --- | --- | --- | --- | --- | --- | --- |
|  | **1** | | | **2** | | | **3 or more** | | |
| **PERIOD 3** | **Cases, n (%)** | **Controls, n (%)** | **OR (95% CI)^a, b^** | **Cases, n (%)** | **Controls, n (%)** | **OR (95% CI)^a, c^** | **Cases, n (%)** | **Controls, n (%)** | **OR (95% CI)^a, d^** |
| **Vaccination status:** | |  |  |  |  |  |  |  |  |
| None | 10089 (45.5) | 3851 (24.9) | 1 | 713 (68.8) | 3579 (33.6) | 1 | 751 (93.6) | 61611 (88.0) | 1 |
| Partly vaccinated | 368 (1.7) | 346 (2.2) | 0.36 (0.29; 0.45) | 26 (2.5) | 222 (2.1) | 0.24 (0.04; 1.51) | 13 (1.6) | 634 (0.9) | 1.02 (0.50; 2.08) |
| Full cycle | 11507 (51.9) | 10237 (66.3) | 0.37 (0.35; 0.39) | 290 (28.0) | 5888 (55.2) | 0.13 (0.08; 0.21) | 37 (4.6) | 6148 (8.8) | 0.39 (0.25; 0.59) |
| Booster dose | 228 (1.0) | 1009 (6.5) | 0.06 (0.05; 0.08) | 8 (0.8) | 975 (9.1) | 0.02 (0.003; 0.07) | 1 (0.1) | 1624 (2.3) | 0.05 (0.01; 0.39) |

^a^Odds Ratio (OR) and 95% confidence intervals (95% CI) adjusted for gender, age group, province of residence and Multisource Comorbidity Score; ^b^The conditional logistic model used 25250 informative observations; ^c^The conditional logistic model used 766 informative observations; ^d^The conditional logistic model used 3169 informative observations.

**Table S3. Frequency and percentage distribution of vaccination status in cases and controls, and multiple conditional logistic regression results stratified by number of swabs performed in the month of the index date. Outcome: COVID-19 infection, PERIOD 4 (20/12/2021-31/3/2022).**

| **PERIOD 4** | **Number of swabs performed in the month of index date:** | | | | | | | | |
| --- | --- | --- | --- | --- | --- | --- | --- | --- | --- |
|  | **1** | | | **2** | | | **3 or more** | | |
|  | **Cases, n (%)** | **Controls, n (%)** | **OR (95% CI)^a, b^** | **Cases**  **n (%)** | **Controls**  **n (%)** | **OR (95% CI)^a, c^** | **Cases**  **n (%)** | **Controls**  **n (%)** | **OR (95% CI)^a, d^** |
| **Vaccination status:** | |  |  |  |  |  |  |  |  |
| None | 36821 (27.2) | 25227 (15.8) | 1 | 3500 (53.6) | 20646 (21.2) | 1 | 4284 (85.4) | 209034 (63.1) | 1 |
| Partly vaccinated | 3296 (2.4) | 4756 (3.0) | 0.46 (0.44; 0.49) | 176 (2.7) | 2741 (2.8) | 0.37 (0.27; 0.51) | 181 (3.6) | 5508 (1.7) | 1.54 (1.24;1.90) |
| Full cycle | 63122 (46.6) | 69617 (43.6) | 0.63 (0.61; 0.64) | 1425 (21.8) | 32949 (33.8) | 0.23 (0.20; 0.26) | 213 (4.2) | 29848 (9.0) | 0.38 (0.32; 0.45) |
| Booster dose | 32285 (23.8) | 60016 (37.6) | 0.36 (0.35; 0.37) | 1430 (21.9) | 41139 (42.2) | 0.16 (0.14; 0.19) | 341 (6.8) | 86815 (26.2) | 0.24 (0.21; 0.27) |

^a^Odds Ratio (OR) and 95% confidence intervals (95% CI) adjusted for gender, age group, province of residence and Multisource Comorbidity Score; ^b^The conditional logistic model used 243689 informative observations; ^c^The conditional logistic model used 7316 informative observations; ^d^The conditional logistic model used 16305 informative observations.

**Table S4. Frequency and percentage distribution of cases and controls characteristics by sub-periods. Outcome: COVID-19-related hospitalization.**

|  | **PERIOD 1** | |  | **PERIOD 2** | |  | **PERIOD 3** | |  | **PERIOD 4** | |
| --- | --- | --- | --- | --- | --- | --- | --- | --- | --- | --- | --- |
|  | **01.02.2021 - 17.05.2021** | |  | **18.5.2021-14.10.2021** | |  | **15.10.2021-19.12.2021** | |  | **20.12.2021-31.3.2022** | |
|  | **Cases** | **Controls** |  | **Cases** | **Controls** |  | **Cases** | **Controls** |  | **Cases** | **Controls** |
|  | **(n=3400)** | **(n=13600)** |  | **(n=429)** | **(n=1716)** |  | **(n=1488)** | **(n=5952)** |  | **(n=2550)** | **(n=10200)** |
| **Characteristic** | **n (%)** | **n (%)** |  | **n (%)** | **n (%)** |  | **n (%)** | **n (%)** |  | **n (%)** | **n (%)** |
| **Gender:** |  |  |  |  |  |  |  |  |  |  |  |
| Female | 1428 (42.0) | 7998 (58.8) |  | 176 (41.0) | 1059 (61.7) |  | 660 (44.4) | 3046 (51.2) |  | 1190 (46.7) | 5458 (53.5) |
| Male | 1972 (580) | 5602 (41.2) |  | 253 (59.0) | 657 (38.3) |  | 828 (55.6) | 2906 (48.8) |  | 1360 (53.3) | 4742 (46.5) |
| **Age group (years):** |  |  |  |  |  |  |  |  |  |  |  |
| 12-19 | 8 (0.2) | 796 (5.9) |  | 2 (0.5) | 162 (9.4) |  | 5 (0.3) | 555 (9.3) |  | 15 (0.6) | 839 (8.2) |
| 20-29 | 24 (0.7) | 1379 (10.1) |  | 21 (4.9) | 181 (10.6) |  | 8 (0.5) | 627 (10.5) |  | 24 (0.9) | 1024 (10.0) |
| 30-39 | 54 (1.6) | 1886 (13.9) |  | 22 (5.1) | 232 (13.5) |  | 42 (2.8) | 924 (15.5) |  | 78 (3.1) | 1481 (14.5) |
| 40-49 | 157 (4.6) | 2830 (20.8) |  | 57 (13.3) | 325 (18.9) |  | 62 (4.2) | 1477 (24.8) |  | 64 (2.5) | 2351 (23.0) |
| 50-59 | 455 (13.4) | 2993 (22.0) |  | 86 (20.0) | 355 (20.7) |  | 169 (11.4) | 1499 (25.2) |  | 208 (8.2) | 2219 (21.8) |
| 60-69 | 629 (18.5) | 1476 (10.9) |  | 64 (14.9) | 175 (10.2) |  | 247 (16.6) | 500 (8.4) |  | 308 (12.1) | 1011 (9.9) |
| 70-79 | 998 (29.4) | 1001 (7.4) |  | 73 (17.0) | 128 (7.5) |  | 402 (27.0) | 174 (2.9) |  | 690 (27.1) | 611 (6.0) |
| 80-89 | 811 (23.9) | 897 (6.6) |  | 71 (16.6) | 117 (6.8) |  | 405 (27.2) | 160 (2.7) |  | 806 (31.6) | 506 (5.0) |
| ≥90 | 264 (7.8) | 342 (2.5) |  | 33 (7.7) | 41 (2.4) |  | 148 (10.0) | 36 (0.6) |  | 357 (14.0) | 158 (1.5) |
| **Province of residence:** |  |  |  |  |  |  |  |  |  |  |  |
| Gorizia | 369 (10.9) | 1807 (13.3) |  | 29 (6.8) | 229 (13.3) |  | 140 (9.4) | 658 (11.1) |  | 303 (11.9) | 1104 (10.8) |
| Pordenone | 420 (12.4) | 1841 (13.53) |  | 108 (25.2) | 254 (14.8) |  | 389 (26.1) | 996 (16.7) |  | 589 (23.1) | 1620 (15.9) |
| Trieste | 657 (19.3) | 3198 (23.53) |  | 162 (37.8) | 412 (24.0) |  | 623 (41.9) | 1371 (23.0) |  | 654 (25.6) | 2135 (20.9) |
| Udine | 1954 (57.5) | 6754 (49.7 |  | 130 (30.3) | 821 (47.8) |  | 336 (22.6) | 2927 (49.2) |  | 1004 (39.4) | 5341 (52.4) |
| **MCS** |  |  |  |  |  |  |  |  |  |  |  |
| 0 | 1135 (33.4) | 8600 (63.2) |  | 183 (42.7) | 116 (65.0) |  | 573 (38.5) | 4433 (74.5) |  | 713 (28.0) | 7128 (69.9) |
| 1-4 | 1085 (31.9) | 3172 (23.3) |  | 134 (31.2) | 371 (11.2) |  | 414 (27.8) | 1206 (20.3) |  | 718 (28.2) | 2209 (21.7) |
| 5-9 | 654 (19.2) | 818 (6.0) |  | 63 (14.7) | 108 (6.3) |  | 289 (19.4) | 164 (2.8) |  | 602 (23.6) | 467 (4.6) |
| 10-14 | 267 (7.9) | 518 (3.8) |  | 30 (7.0) | 71 (4.1) |  | 123 (8.3) | 80 (1.3) |  | 257 (10.1) | 223 (2.2) |
| 15-19 | 136 (4.0) | 209 (1.5) |  | 8 (1.9) | 31 (1.8) |  | 51 (3.4) | 36 (0.6) |  | 139 (5.5) | 90 (0.9) |
| ≥20 | 123 (3.6) | 283 (2.1) |  | 11 (2.6) | 19 (1.1) |  | 38 (2.6) | 33 (0.6) |  | 121 (4.8) | 83 (0.8) |
| **Vaccination status:** |  |  |  |  |  |  |  |  |  |  |  |
| None | 3269 (96.2) | 9707 (71.4) |  | 292 (68.1) | 827 (48.2) |  | 803 (54.0) | 4364 (73.3) |  | 931 (36.5) | 4629 (45.4) |
| Partly vaccinated | 105 (3.1) | 966 (7.1) |  | 15 (3.5) | 102 (5.9) |  | 24 (1.6) | 60 (1.0) |  | 79 (3.1) | 224 (2.2) |
| Full cycle | 26 (0.8) | 2927 (21.5) |  | 122 (28.4) | 787 (45.8) |  | 635 (42.7) | 1333 (22.4 |  | 810 (31.8) | 2182 (21.4) |
| Booster dose | 0 (0.0) | 0 (0.0) |  | 0 (0.0) | 0 (0.0) |  | 26 (1.8) | 195 (3.3) |  | 730 (28.6) | 3165 (31.0) |
| **Swabs in index-date’s month^,a,b^:** |  |  |  |  |  |  |  |  |  |  |  |
| 1 | 3287 (96.7) | 6216 (45.7) |  | 412 (96.0) | 738 (43.0) |  | 1377 (92.5) | 916 (15.4) |  | 2343 (91.9) | 2668 (26.2) |
| 2 | 73 (2.2) | 3347 (24.6) |  | 14 (3.3) | 386 (22.5) |  | 74 (5.0) | 623 (10.5) |  | 93 (3.6) | 1581 (15.5) |
| ≥3 | 40 (1.2) | 4037 (29.7) |  | 3 (0.7) | 592 (34.5) |  | 37 (2.5) | 4413 (74.1) |  | 114 (4.5) | 5951 (58.3) |
| ^a^Number of swabs performed in the month of index date  ^b^Case’s date of sampling matched with control’s date of sampling. | | | | | | | | | | | |

**Table S5. Frequency and percentage distribution of vaccination status in cases and controls, and multiple conditional logistic regression results stratified by number of swabs performed in the month of the index date. Outcome: COVID-19-related hospitalization, PERIOD 3 (15/10/2021-19/12/2021).**

|  | **Number of swabs performed in the month of index date:** | | | | | | | | |
| --- | --- | --- | --- | --- | --- | --- | --- | --- | --- |
|  | **1** | | | **2** | | | **3 or more** | | |
| **PERIOD 3** | **Cases, n (%)** | **Controls, n (%)** | **OR (95% CI)^a, b^** | **Cases, n (%)** | **Controls, n (%)** | **OR (95% CI)^a, c^** | **Cases, n (%)** | **Controls, n (%)** | **OR (95% CI)^a, d^** |
| **Vaccination status:** | |  |  |  |  |  |  |  |  |
| None | 740 (53.7) | 237 (25.9) | 1 | 35 (47.3) | 224 (36.0) | 1 | 28 (75.7) | 3903 (88.4) | 1 |
| Partly vaccinated | 20 (1.5) | 21 (2.3) | 0.23 (0.04; 1.22) | 2 (2.7) | 9 (1.4) | NE^e^ | 2 (5.4) | 30 (0.7) | 1.23 (0.02; 76.26) |
| Full cycle | 593 (43.1) | 609 (66.5) | 0.09 (0.05; 0.16) | 35 (47.3) | 332 (53.3) | NE^e^ | 7 (18.9) | 392 (8.9) | 1.82 (0.03; 112.36) |
| Booster dose | 24 (1.7) | 49 (5.3) | 0.004 (<0.001; 0.02) | 2 (2.7) | 58 (9.3) | NE^e^ | 0 (0.0) | 88 (2.0) | - |

^a^Odds Ratio (OR) and 95% confidence intervals (95% CI) adjusted for gender, age group, province of residence and Multisource Comorbidity Score; ^b^The conditional logistic model used 1530 informative observations; ^c^The conditional logistic model used 67 informative observations.; ^d^The conditional logistic model used 144 informative observations; ^e^The OR were not estimated (NE) because Newton-Raphson Ridge Optimization cannot be completed.

**Table S6. Frequency and percentage distribution of vaccination status in cases and controls, and multiple conditional logistic regression results stratified by number of swabs performed in the month of the index date. Outcome: COVID-19-related hospitalization, PERIOD 4 (20/12/2021-31/3/2022).**

| **PERIOD 4** | **Number of swabs performed in the month of index date:** | | | | | | | | | |
| --- | --- | --- | --- | --- | --- | --- | --- | --- | --- | --- |
|  | **1** | | | | **2** | | | **3 or more** | | |
|  | **Cases, n (%)** | **Controls, n (%)** | **OR (95% CI)^a, b^** | **Cases, n (%)** | | **Controls, n (%)** | **OR (95% CI)^a, c^** | **Cases, n (%)** | **Controls, n (%)** | **OR (95% CI)^a, d^** |
| **Vaccination status:** | |  |  |  | |  |  |  |  |  |
| None | 849 (17.2) | 458 (17.2) | 1 | 33 (35.5) | | 334 (21.1) | 1 | 49 (43.0) | 3837 (64.5) | 1 |
| Partly vaccinated | 66 (2.8) | 74 (2.8) | 0.42 (0.20; 0.86) | 3 (3.2) | | 49 (3.1) | 0.40 (<0.001; >999) | 10 (8.8) | 101 (1.7) | >999 (<0.001; >999) |
| Full cycle | 749 (31.9) | 1127 (42.2) | 0.31 (0.24; 0.42) | 28 (30.1) | | 539 (34.1) | 0.54 (0.10; 2.85) | 34 (29.8) | 516 (8.7) | 1.75 (0.53; 5.77) |
| Booster dose | 680 (29.0) | 1009 (37.8) | 0.10 (0.07; 0.14) | 29 (31.2) | | 659 (41.7) | 0.23 (0.03; 2.08) | 21 (18.4) | 1497 (25.2) | 0.09 (0.02; 0.38) |

^a^Odds Ratio (OR) and 95% confidence intervals (95% CI) adjusted for gender, age group, province of residence and Multisource Comorbidity Score; ^b^The conditional logistic model used 4048 informative observations; ^c^The conditional logistic model used 128 informative observations; ^d^The conditional logistic model used 360 informative observations.

**Table S7. Frequency and percentage distribution of cases and controls characteristics by sub-periods. Outcome: COVID-19-related death**

|  | **PERIOD 1** | |  | **PERIOD 2** | |  | **PERIOD 3** | |  | **PERIOD 4** | |
| --- | --- | --- | --- | --- | --- | --- | --- | --- | --- | --- | --- |
|  | **01.02.2021 - 17.05.2021** | |  | **18.5.2021-14.10.2021** | |  | **15.10.2021-19.12.2021** | |  | **20.12.2021-31.3.2022** | |
|  | **Cases** | **Controls** |  | **Cases** | **Controls** |  | **Cases** | **Controls** |  | **Cases** | **Controls** |
|  | **(n=915)** | **(n=3660)** |  | **(n=58)** | **(n=232)** |  | **(n=322)** | **(n=1288)** |  | **(n=915)** | **(n=3660)** |
| **Characteristic** | **n (%)** | **n (%)** |  | **n (%)** | **n (%)** |  | **n %** | **n %** |  | **n %** | **n %** |
| **Gender:** |  |  |  |  |  |  |  |  |  |  |  |
| Female | 386 (42.2) | 2104 (57.5) |  | 24 (41.4) | 142 (61.2) |  | 145 (45.0) | 627 (48.7) |  | 344 (46.2) | 1602 (53.8) |
| Male | 529 (57.8) | 1556 (42.5) |  | 34 (58.6) | 90 (38.8) |  | 177 (55.0) | 661 (51.3) |  | 401 (53.8) | 1378 (46.2) |
| **Age group (years):** |  |  |  |  |  |  |  |  |  |  |  |
| 12-19 | 0 (0.0) | 189 (5.2) |  | 0 (0.0) | 22 (9.5) |  | 0 (0.0) | 116 (9.0) |  | 1 (0.1) | 260 (8.7) |
| 20-29 | 0 (0.0) | 349 (9.5) |  | 0 (0.0) | 30 (12.9) |  | 0 (0.0) | 150 (11.6) |  | 0 (0.0) | 283 (9.5) |
| 30-39 | 0 (0.0) | 544 (14.9) |  | 0 (0.0) | 36 (15.5) |  | 0 (0.0) | 216 (16.8) |  | 0 (0.0) | 430 (14.4) |
| 40-49 | 3 (0.3) | 744 (20.3) |  | 1 (1.7) | 39 (16.8) |  | 2 (0.6) | 286 (22.2) |  | 4 (0.5) | 678 (22.8) |
| 50-59 | 23 (2.5) | 820 (22.4) |  | 2 (3.4) | 41 (17.7) |  | 7 (2.2) | 350 (27.2) |  | 14 (1.9) | 624 (20.9) |
| 60-69 | 87 (9.5) | 388 (10.6) |  | 3 (5.2) | 21 (9.1) |  | 23 (7.1) | 96 (7.5) |  | 55 (7.4) | 325 (10.9) |
| 70-79 | 270 (29.5) | 299 (8.2) |  | 15 (25.9) | 26 (11.2) |  | 82 (25.5) | 31 (2.4) |  | 168 (22.6) | 197 (6.6) |
| 80-89 | 359 (39.2) | 230 (6.3) |  | 20 (34.5) | 14 (6.0) |  | 121 (37.6) | 33 (2.6) |  | 304 (40.8) | 133 (4.5) |
| ≥90 | 173 (18.9) | 97 (2.7) |  | 17 (29.3) | 3 (1.3) |  | 87 (27.0) | 10 (0.8) |  | 199 (26.7) | 50 (1.7) |
| **Province of residence:** |  |  |  |  |  |  |  |  |  |  |  |
| Gorizia | 93 (10.1) | 465 (12.7) |  | 7 (12.1) | 27 (11.6) |  | 44 (13.7) | 153 (11.9) |  | 101 (13.6) | 305 (10.2) |
| Pordenone | 79 (8.6) | 454 (12.4) |  | 11 (19.0) | 37 (15.9) |  | 81 (25.2) | 200 (15.5) |  | 167 (22.4) | 495 (16.6) |
| Trieste | 164 (17.9) | 920 (25.1) |  | 25 (43.1) | 57 (24.6) |  | 159 (49.4) | 288 (22.4) |  | 220 (29.5) | 650 (21.8) |
| Udine | 579 (63.3) | 1821 (49.8) |  | 15 (25.9) | 111 (47.8) |  | 38 (11.8) | 647 (50.2) |  | 257 (34.5) | 1530 (51.3) |
| **MCS** |  |  |  |  |  |  |  |  |  |  |  |
| 0 | 176 (19.2) | 2275 (62.2) |  | 10 (17.2) | 163 (70.3) |  | 63 (19.6) | 966 (75.0) |  | 166 (22.3) | 2050 (68.8) |
| 1-4 | 248 (27.1) | 858 (23.4) |  | 15 (25.9) | 39 (16.8) |  | 75 (23.3) | 262 (20.3) |  | 179 (24.0) | 663 (22.2) |
| 5-9 | 256 (28.0) | 241 (6.6) |  | 15 (25.9) | 14 (6.0) |  | 104 (32.3) | 39 (3.0) |  | 194 (26.0) | 129 (4.3) |
| 10-14 | 114 (12.5) | 131 (3.6) |  | 6 (10.3) | 9 (3.9) |  | 43 (13.4) | 13 (1.0) |  | 107 (14.4) | 73 (2.4) |
| 15-19 | 56 (6.1) | 68 (1.9) |  | 4 (6.9) | 2 (0.9) |  | 20 (6.2) | 6 (0.5) |  | 48 (6.4) | 37 (1.2) |
| ≥20 | 65 (7.1) | 87 (2.4) |  | 8 (13.8) | 5 (2.2) |  | 17 (5.3) | 2 (0.2) |  | 51 (6.8) | 28 (0.9) |
| **Vaccination status:** |  |  |  |  |  |  |  |  |  |  |  |
| None | 836 (91.4) | 2518 (68.8) |  | 28 (48.3) | 98 (42.2) |  | 131 (40.7) | 942 (73.1) |  | 275 (36.9) | 1340 (45.0) |
| Partly vaccinated | 71 (7.8) | 232 (6.3) |  | 5 (8.6) | 18 (7.8) |  | 9 (2.8) | 15 (1.2) |  | 24 (3.2) | 75 (2.5) |
| Full cycle | 8 (0.9) | 910 (24.9) |  | 25 (43.1) | 116 (50.0) |  | 164 (50.9) | 284 (22.1) |  | 219 (29.4) | 614 (20.6) |
| Booster dose | 0 (0.0) | 0 (0.0) |  | 0 (0.0) | 0 (0.0) |  | 18 (5.6) | 47 (3.6) |  | 227 (30.5) | 951 (31.9) |
| **Swabs in index-date’s month^a,b^:** |  |  |  |  |  |  |  |  |  |  |  |
| 1 | 873 (95.4) | 1652 (45.1) |  | 55 (94.8) | 108 (46.6) |  | 288 (89.4) | 183 (14.2) |  | 685 (91.9) | 764 (25.6) |
| 2 | 25 (2.7) | 899 (24.6) |  | 1 (1.7) | 44 (19.0) |  | 25 (7.8) | 133 (10.3) |  | 30 (4.0) | 440 (14.8) |
| ≥3 | 17 (1.9) | 1109 (30.3) |  | 2 (3.5) | 80 (34.5) |  | 9 (2.8) | 972 (75.5) |  | 30 (4.0) | 1776 (59.6) |
| ^a^Number of swabs performed in the month of index date  ^b^Case’s date of death matched with control’s date of sampling. | | | | | | | | | | | |

**Table S8. Frequency and percentage distribution of vaccination status in cases and controls, and multiple conditional logistic regression results stratified by number of swabs performed in the month of the index date. Outcome: COVID-19-related death, PERIOD 3 (15/10/2021-19/12/2021).**

|  | **Number of swabs performed in the month of index date:** | | | | | | | | |
| --- | --- | --- | --- | --- | --- | --- | --- | --- | --- |
|  | **1** | | | **2** | | | **3 or more** | | |
| **PERIOD 3** | **Cases, n (%)** | **Controls, n (%)** | **OR (95% CI)^a, b^** | **Cases, n (%)** | **Controls, n (%)** | **OR (95% CI)^a, c^** | **Cases, n (%)** | **Controls, n (%)** | **OR (95% CI)^a, d^** |
| **Vaccination status:** | |  |  |  |  |  |  |  |  |
| None | 121 (42.0) | 46 (25.1) | 1 | 6 (24.0) | 55 (41.4) | 1 | 4 (44.4) | 841 (86.5) | 1 |
| Partly vaccinated | 8 (2.8) | 4 (2.2) | 0.29 (0.15; 0.58) | 1 (4.0) | 4 (3.0) | NE^e^ | 5 (55.6) | 7 (07) | NE^e^ |
| Full cycle | 142 (49.3) | 118 (64.5) | 0.13 (0.08; 0.21) | 17 (68.0) | 62 (46.6) | NE^e^ | 0 (0.0) | 104 (10.7) | NE^e^ |
| Booster dose | 17 (5.9) | 15 (8.2) | 0.06 (0.03; 0.12) | 1 (4.0) | 12 (9.0) | NE^e^ | 0 (0.0) | 20 (2.1) | NE^e^ |

^a^Odds Ratio (OR) and 95% confidence intervals (95% CI) adjusted for gender, age group, province of residence and Multisource Comorbidity Score. ^b^The conditional logistic model used 4011 informative observations; ^c^The conditional logistic model used 99 informative observations; ^d^The conditional logistic model used 196 informative observations; ^e^The OR were not estimated (NE) because Newton-Raphson Ridge Optimization cannot be completed.

**Table S9. Frequency and percentage distribution of vaccination status in cases and controls, and multiple conditional logistic regression results stratified by number of swabs performed in the month of the index date. Outcome: COVID-19-related death, PERIOD 4 (20/12/2021-31/3/2022).**

| **PERIOD 4** | **Number of swabs performed in the month of index date:** | | | | | | | | | |
| --- | --- | --- | --- | --- | --- | --- | --- | --- | --- | --- |
|  | **1** | | | **2** | | | **3 or more** | | | |
|  | **Cases, n (%)** | **Controls, n (%)** | **OR (95% CI)^a, b^** | **Cases, n (%)** | **Controls, n (%)** | **OR (95% CI)^a, c^** | **Cases, n (%)** | **Controls, n (%)** | **OR (95% CI)^a, d^** |  |
| **Vaccination status:** | |  |  |  |  |  |  |  |  |  |
| None | 258 (37.7) | 137 (17.9) | 1 | 5 (16.7) | 94 (21.4) | 1 | 12 (40.0) | 1109 (62.4) | 1 |  |
| Partly vaccinated | 21 (3.1) | 25 (3.3) | 0.25 (0.03; 2.37) | 0 (0.0) | 22 (5.0) | NE^e^ | 3 (10.0) | 28 (1.6) | NE^e^ |  |
| Full cycle | 195 (28.5) | 303 (39.7) | 0.31 (0.14; 0.67) | 16 (53.3) | 138 (31.4) | NE^e^ | 8 (26.7) | 173 (9.7) | NE^e^ |  |
| Booster dose | 211 (30.8) | 299 (39.1) | 0.09 (0.04; 0.22) | 9 (30.0) | 186 (42.3) | NE^e^ | 7 (23.3) | 466 (26.2) | NE^e^ |  |

^a^Odds Ratio (OR) and 95% confidence intervals (95% CI) adjusted for gender, age group, province of residence and Multisource Comorbidity Score; ^c^The conditional logistic model used 26 informative observations; ^d^The conditional logistic model used 110 informative observations; ^e^The OR were not estimated (NE) because Newton-Raphson Ridge Optimization cannot be completed.

**Table S10. Comparison of our results with the meta-analysis from Shao et al. [30].**

|  | **Vaccination status** | **VE vs Infection**  **(95% CI)** | **N.**  **studies** | **VE vs Hospitalization**  **(95% CI)** | **N.**  **studies** | **VE vs Death**  **(95% CI)** | **N.**  **studies** |
| --- | --- | --- | --- | --- | --- | --- | --- |
| **ALFA VOC** |  |  |  |  |  |  |  |
| Shao et al. (7) | Partial_(summary)_ | 46.1% (34.2, 55.8) | 27 | 69.9% (59.2, 77.7) | 10 | 66.8% (52.7, 76.7) | 5 |
| Our study | Partial | 77% (75, 78) |  | 76% (70, 82) |  | 62% (39, 76) |  |
| Shao et al. (7) | Full_(gen pop)_ | 89.6% (85.1, 92.8) | 18 | 92.1% (82.9, 96.4) | 8 | 95.7% (87.7, 98.5) | 4 |
| Our study | Full | 96% (96, 97) |  | 97% (95, 98) |  | 98% (94, 99) |  |
| **DELTA VOC** |  |  |  |  |  |  |  |
| Shao et al. (7) | Partial_(summary)_ | 50.0% (46.9, 52.9) | 25 | 68.3% (51.2, 79.4) | 10 | 89.2% (84.2, 92.6) | 3 |
| Our study | Partial | 26% (17, 34)  58% (5) |  | 76% (53, 88)  13% (<02) |  | 95% (<0, 99.9)  <0 (<0 - 9) |  |
| Shao et al. (7) | Full_(summary)_ | 70.9% (68.9, 72.7) | 43 | 84.9% (82.4, 87.1) | 24 | 90.3% (82.4, 94.7) | 4 |
| Our study | Full | 55% (52, 58)  65% (63 - 67) |  | 84% (77, 89)  87% (80 91) |  | 98% (72, 99.99)  94% (4 - 99) |  |
| Shao et al. (7) | Booster_(summary)_ | 93.3% (91.7, 94.6) | 7 | 92.8% (89.1, 95.2) | 11 |  |  |
| Our study | Booster | 94% (93, 95) |  | 98% (95, 99) |  | 99.9% (29, >99) |  |
| **OMICRON VOC** |  |  |  |  |  |  |  |
| Shao et al. (7) | Partial_(summary)_ | 25.9% (20.0, 34.9) | 6 |  |  |  |  |
| Our study | Partial | 52% (50, 54) |  | 20% (<0, 54) |  | 31% (<0, 83) |  |
| Shao et al. (7) | Full_(summary)_ | 23.5% (17.0, 29.5) | 12 | 56.5% ( 50.9, 61.4) | 12 | 82.4% (66.1, 90.9) | 3 |
| Our study | Full | 43% (42, 45) |  | 59% (49, 66) |  | 63% (31, 80) |  |
| Shao et al. (7) | Booster_(summary)_ | 57.6% (55.1, 59.9) | 12 | 83.4% (80.7, 85.8) | 10 | 94.9% (89.2, 97.6) | 3 |
| Our study | Booster | 67% (66, 67) |  | 87% (83, 90) |  | 90% (82, 95) |  |
